# Working memory training improves emotional states of healthy individuals

**DOI:** 10.3389/fnsys.2014.00200

**Published:** 2014-10-16

**Authors:** Hikaru Takeuchi, Yasuyuki Taki, Rui Nouchi, Hiroshi Hashizume, Atsushi Sekiguchi, Yuka Kotozaki, Seishu Nakagawa, Carlos Makoto Miyauchi, Yuko Sassa, Ryuta Kawashima

**Affiliations:** ^1^Division of Developmental Cognitive Neuroscience, Institute of Development, Aging and Cancer, Tohoku UniversitySendai, Japan; ^2^Division of Medical Image Analysis, Department of Community Medical Supports, Tohoku Medical Megabank Organization, Tohoku UniversitySendai, Japan; ^3^Department of Radiology and Nuclear Medicine, Institute of Development, Aging and Cancer, Tohoku UniversitySendai, Japan; ^4^Department of Advanced Brain Science, Smart Ageing International Research Center, Institute of Development, Aging and Cancer, Tohoku UniversitySendai, Japan; ^5^Department of Functional Brain Imaging, Institute of Development, Aging and Cancer, Tohoku UniversitySendai, Japan

**Keywords:** working memory, training, plasticity, emotion, mood, anger, fMRI

## Abstract

Working memory (WM) capacity is associated with various emotional aspects, including states of depression and stress, reactions to emotional stimuli, and regulatory behaviors. We have previously investigated the effects of WM training (WMT) on cognitive functions and brain structures. However, the effects of WMT on emotional states and related neural mechanisms among healthy young adults remain unknown. In the present study, we investigated these effects in young adults who underwent WMT or received no intervention for 4 weeks. Before and after the intervention, subjects completed self-report questionnaires related to their emotional states and underwent scanning sessions in which brain activities related to negative emotions were measured. Compared with controls, subjects who underwent WMT showed reduced anger, fatigue, and depression. Furthermore, WMT reduced activity in the left posterior insula during tasks evoking negative emotion, which was related to anger. It also reduced activity in the left frontoparietal area. These findings show that WMT can reduce negative mood and provide new insight into the clinical applications of WMT, at least among subjects with preclinical-level conditions.

## Introduction

Working memory (WM) is the limited capacity storage system involved in the maintenance and manipulation of information over a short time period (Baddeley, 2003). It is a functionally important system that underlies a wide range of higher-order cognitive activities such as general intelligence, reasoning and problem solving, language comprehension, learning, and response inhibition (Osaka and Nishizaki, 2000; Baddeley, 2003). Reduced WM capacity (WMC) is also associated with a wide variety of emotional aspects, including mood disorders, anxiety, stress, greater emotional responses, and fewer regulatory behaviors (Sorg and Whitney, 1992; Klein and Boals, 2001; Weiland-Fiedler et al., 2004; Schmeichel et al., 2008).

The dorsolateral prefrontal cortex (DLPFC) plays a key role in the central executive system of WM (Baddeley, 2003). The premotor cortex is closely related to DLPFC, both anatomically, and functionally (Fuster, 2006; Takeuchi et al., 2012), and it plays important roles in some executive functions of WM (Wager et al., 2008). Both areas are consistently active during the execution of WM (Reuter-Lorenz et al., 2000). They also play important roles in emotional aspects, and an essential link exists between DLPFC to premotor areas and mood as described below. Functional deficits of DLPFC to dorsal premotor areas are associated with depression (Siegle et al., 2007). DLPFC to premotor areas and other widespread lateral prefrontal and medial prefrontal regions are activated to control negative emotions (Phan et al., 2005; Belden et al., 2014). Rapid-rate transcranial magnetic stimulation (rTMS) over DLPFC as well as premotor areas mitigates depression (Pascual-Leone et al., 1996; Johnson et al., 2013). Activity changes in DLPFC to dorsal premotor areas and the precentral gyrus have been associated with depression (Koenigs and Grafman, 2009; Stuhrmann et al., 2011). DLPFC lesions have been shown to cause apathy and greater vulnerability to fatigue and depression compared with other lesions (Fuster, 2006; Koenigs and Grafman, 2009), and structural abnormalities in DLPFC to premotor areas are associated with fatigue (Chaudhuri and Behan, 2004).

Physical exercise can affect the volume of DLPFC to premotor areas (Colcombe et al., 2004, 2006; Flöel et al., 2010) and improve the cognitive functions that are related to DLPFC to premotor areas (Smith et al., 2010); further, it can robustly improve mood (Arent and Landers, 2000). Other studies have shown that WM training (WMT) can affect the outcomes of psychological measures and neural systems (Uchida and Kawashima, 2008; Klingberg, 2010; Takeuchi et al., 2010b). However, whether WMT can improve cognitive performance is still a matter of debate because several studies have reported different conclusions (Jaeggi et al., 2008; Redick et al., 2013). A recent meta-analysis indicated that WMT has robust effects on WMC and measures of inhibition and attention (Melby-Lervåg and Hulme, 2012). In addition, WMT has been clinically shown to improve fatigue in patients with multiple sclerosis (Takeuchi et al., 2010b) as well as the symptoms of attention deficit hyperactivity disorder (Klingberg et al., 2005), which can affect mood (e.g., impulsivity can lead to a failure in emotional regulation) (Apter et al., 1990). Structural and functional studies that have used magnetic resonance imaging (MRI), positron emission tomography (PET), and near infrared spectroscopy (NIRS), have linked WMT to changes in brain activity during WM and other related tasks, changes in gray and white matter structures, and dopamine D1 receptor density in prefrontal (including DLPFC and premoror) and posterior parietal areas (McNab et al., 2009; Klingberg, 2010; Takeuchi et al., 2010b, 2013b, 2014a; Buschkuehl et al., 2012; McKendrick et al., 2014). In summary, as described, TMS applied to DLPFC to premotor areas improves mood, whereas DLPFC lesions can deteriorate mood. Similarly, deteriorated emotional states have been associated with hypoactivity in DLPFC to premotor areas. Other interventions such as aerobic exercise can improve the structure and functioning of DLPFC to premotor areas and subsequently improve mood (Lawlor and Hopker, 2001). WMT leads to changes in several mechanisms of DLPFC to premotor areas and cognitive functions related to the areas (Takeuchi et al., 2010b, 2013b). Further, emotional states can be changed through a wide range of interventions in both clinical and nonclinical samples (McNair et al., 1992).

These previous findings have led us to question whether WMT can improve emotional states in individuals without apparent cognitive deficits. We investigated this question in the present study in young adults who underwent WMT. The subjects completed self-report questionnaires related to their emotional states before and after WMT and were examined with a functional MRI (fMRI) task to detect negative emotion-related brain activity as they identified faces with negative emotions (angry or afraid) (Hariri et al., 2002). Previously, this task has been used to examine whether individuals at risk for emotional problems (e.g., subjects with a genetic risk for impulsivity and violence) show different activity in emotion-related areas such as the anterior insula (Meyer-Lindenberg et al., 2006). Our primary aim was to investigate the effects of WMT on mood and its underlying neural activity. We believe that there is no evidence that indicates that improvements in emotional states are solely produced by increased emotional regulation. Considering that our emotional states contribute to our everyday well-being, it is important to investigate the extent of their plasticity and develop new methods to improve them, which could lead to the clinical application of WMT. We hypothesized that WMT improves an emotional state and alters neural activity related to negative emotions, particularly in DLPFC to premotor areas and areas related to emotional states. The increase or decrease in task-induced activity after training or interventions depends on many factors. Even when training leads to adaptations in performance or neural mechanisms, a training-related increase in the efficiency of the involved areas (hence, an activity decrease) or a training-related increase in recruitment of the critical areas (hence, an activity increase) may underlie these changes (Erickson et al., 2007). Thus, we did not predict the direction (increase or decrease) of changes.

## Materials and methods

### Subjects

#### Subjects characteristics in each group and experiment

**Figure 1:**
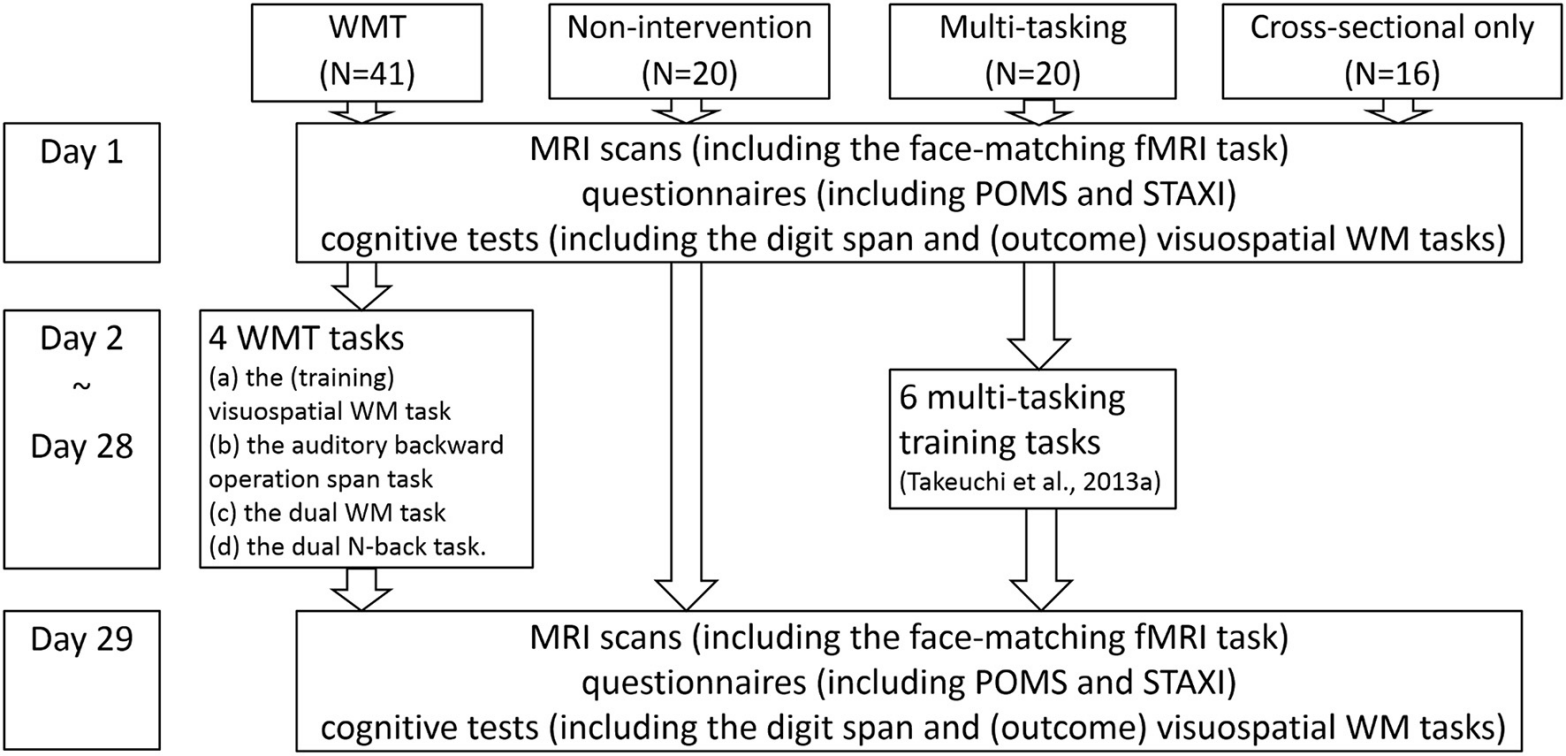
**The schema of this study's groups, tasks, and experimental design**. A figure illustrating subject flow can be seen in our previous report (Takeuchi et al., 2014b). The measurements for day 1 and day 29 are in no particular order.

We recruited 97 students and graduates to participate in experiments in our laboratory. Of these 97 subjects, 81 were included in the longitudinal intervention experiment, which involved two intervention studies and three groups (a WMT group, another intervention group for another longitudinal study, and a non-intervention control group, Figure 1). The remaining 16 subjects were included in a cross-sectional experiment that examined brain activity during a face-matching task and completed only all other preintervention procedures [16 women; mean age = 21.6 years; standard deviation (*SD*) = 1.9; age range = 19–27]. Among the 81 subjects included in the longitudinal intervention experiment, 61 were assigned to the WMT or non-intervention control group. The WMT group consisted of 41 subjects (27 men, 14 women) with a mean age of 20.9 years (*SD* = 1.6; age range = 18–24). The non-intervention group consisted of 20 subjects (15 men, 5 women) with a mean age of 21.4 years (*SD* = 2.2; age range = 18–26). Among the 81 subjects included in the longitudinal intervention experiment, the remaining 20 subjects were assigned to a second intervention group (multitasking training group) which is irrelevant to the purpose of this study. Details of this intervention have been described elsewhere (Takeuchi et al., 2014b).

The WMT and control groups were initially similar (*P* > 0.1, two-tailed *t*-tests) in terms of age, sex, general intelligence scores, WM measures (visuospatial WM and digit span tasks) that were used as outcome measures (Takeuchi et al., 2013b), or mood measures used in this study. Subjects who could not be followed throughout the study because of illness (one subject in the control group) or who failed to submit the required data [one subject in the WMT group did not submit the postintervention Profile of Mood States (POMS) data] were excluded from the relevant analyses. For the description of the subjects flow in the present longitudinal experiment of 81 subjects, please see the figure from our previous report (Takeuchi et al., 2014b).

#### Subjects' inclusion and exclusion criteria

The study participants were college students at Tohoku University or graduates from the university in the previous year, who were recruited with advertisements posted on bulletin boards or via email, both of which specified the inclusion and exclusion criteria for participation in the study. The exclusion criteria included left-handedness, implanted metal devices, metal around the body, claustrophobia, the use of certain drugs, a history of psychiatric or neurological diseases, not having a laptop personal computer running Windows (Microsoft, USA), and previous participation in related experiments. We provided questionnaires to all potential participants to assess for psychiatric illnesses and to request disclosure of recent drug use. None of the patients reported a history of neurological or psychiatric illness. These questionnaires were administered during the pre-experiment explanation and based on voluntary self-report. Handedness was evaluated using the Edinburgh Handedness Inventory (Oldfield, 1971). All the subjects had normal vision.

#### Group assignment of participants and experimental periods

Group assignments were performed in a non-arbitrary manner. As described in our previous study (Takeuchi et al., 2013b), groups of participants completed the preintervention- and postinterevention-MRI studies and psychological experiments during different predetermined experimental periods (for example, one group participated in the periods starting 4 weeks from November 4th, another group participated in the periods starting 4 weeks from November 10th, and so on). Participants in one predetermined experimental period were randomly assigned to one of the two groups (e.g., the WMT group or the non-intervention control group). The two groups assigned were different for each predetermined experimental period. This means, for example, that participants in the 4-week periods starting from November 4th and November 25th were assigned to the WMT group or the non-intervention control group, but participants in the 4-week periods starting from November 10th and September 2nd were assigned to the MT training group or the non-intervention control group, yet participants in the 4-week periods starting from November 16th and September 10th were assigned to the WMT group or the MT training group. The participants selected the period they wished to participate in and were not notified that there were two intervention groups before the experiment. Which periods have which two groups are ordered in non-specific manner. Thus, basically, subjects were supposed to be assigned to one of three groups without any bias in a non-arbitrary manner.

#### The purpose of this study and those of other studies using the data of WMT groups in this study

In this experiment, we aimed to investigate a wide range of distinct research topics, including the effects of polymorphism on cognitive training. We previously investigated the effects of WMT on resting-state FC, regional cerebral blood flow at rest (resting-rCBF), and gray matter structure as well as performance on cognitive tests (Takeuchi et al., 2013b), none of which overlap the measures investigated in the present study. Given these diverse aims and the possible infinite sources of variation, we focused on the effect of WMT on emotional states. More subjects were included in the WMT group to facilitate analysis of polymorphism in WMT, as described above.

#### Ethical issues

Written informed consent was obtained from each individual for the projects in which they participated. The Ethics Committee of Tohoku University approved all procedures.

### Procedure

#### General procedures of WMT

The WMT task included four computerized tasks that were Borland C++ programs developed in-house. Subjects trained in the task 20–60 min a day for 27 days. We chose a 4-week intervention period because the duration of WMT programs is often 4–5 weeks (Takeuchi et al., 2010b). However, the total training time depended on the level and the time between trials. The length of each training period varied because we did not limit training by time to prevent subjects from finishing the training without completing the tasks properly. Instead, task completion was based on the number of correctly completed trials (in other words, we controlled the length of the tasks based on how many trials each subject completed the tasks correctly). Thus, subjects could not finish the tasks without completing them correctly no matter how much time they spent on them. This means that if the subject's condition was poor, they took longer to complete the training of the day. However, heterogeneity in the amount of overall training among subjects was unlikely because when the subjects recovered the following day they would be able to finish the tasks more quickly (because the level of the tasks would have dropped when the subject's condition was poor). Subjects performed the tasks on their personal computers and were advised to perform the WMT task daily, with two training sessions a week conducted in the laboratory. A log that recorded task performance and the time when subjects completed each trial was used to determine the date and duration of training sessions. Further details can be found in our previous study (Takeuchi et al., 2013b). For the reasons why we used the non-intervention group as a control group, and possible consideration of placebo effects, please see Supplemental Discussion.

#### Monetary reward

Subjects including those of the control group, received a monetary reward based on the extent to which they participated in the experiments. For training period, to strictly control the amount of training and to motivate subjects to undertake regular training, (a) the subjects received a monetary reward for the training part of the experiment on the basis of the number of sessions they completed; however, (b) they were not given a monetary reward for excessive training (>27 sessions), and (c) the maximum amount of monetary reward was received when one training session was completed each day, even if 27 training sessions were completed. If multiple training sessions were performed in a day, the monetary reward for any second and subsequent training sessions was reduced. For the consideration effects of monetary rewards on moods, see Supplemental Discussion. Basically we failed to find such effects as monetary reward inducement of mood change after the intervention period in this kind of experiment.

### Training tasks

The WMT task is described in our previous report (Takeuchi et al., 2013b). In brief, four WMT tasks were presented during a single training session. In each task, the difficulty (number of items to be remembered) level was modulated on the basis of the subject's performance. Four training tasks were used because increasing the task variability, stimuli, and training situations leads to a more successful transfer (Sweller et al., 1998; Yamnill and McLean, 2001; Green and Bavelier, 2008). The four WMT tasks were as follows: (a) a visuospatial WM task, (b) an auditory backward operation span task, (c) a dual WM task, and (d) a dual N-back task. Additional details are presented in Supplemental Methods. Note that the visuospatial WM task for WMT was different from that used as the outcome measure (Takeuchi et al., 2013b).

We quantified the subjects' performance on all trained WM tasks to analyze their training-related changes in the WM tasks (please see our previous study for details; Takeuchi et al., 2013b). The difference between the composite scores for the first three sessions and the last three sessions was represented as performance changes in the WMT tasks. This method was used in previous studies (Takeuchi et al., 2010a, 2013b, 2014a,b) because it provides a stable performance measure for multiple tasks across three training days.

### Psychological outcome measures

Neuropsychological tests and questionnaires were administered for preintervention and postintervention evaluation.

#### POMS

The shortened Japanese version (Yokoyama, 2005) of the POMS (McNair et al., 1992) questionnaire was used to measure a participant's mood on the day of the experiment as well as in the preceding week. The POMS questionnaire consists of six individual subscales: tension/anxiety, depression/dejection, anger/hostility, vigor/activity, fatigue/inertia, and confusion/bewilderment. We used the POMS subscale score for the preceding week to investigate whether WMT improved emotional states. Since its release, POMS has proven to be an excellent measure of fluctuations in affective mood states across a broad population, including psychiatric outpatients, medical patients, and sports subjects (McNair et al., 1992). POMS identifies and assesses transient, fluctuating affective mood states (McNair et al., 1992). It can be administered on a weekly basis, which is a sufficient period for detecting the respondent's mood responses to his or her current life situation but still short enough to assess acute treatment effects (McNair et al., 1992).

#### State anger scale of the state–trait anger expression inventory

The State Anger scale of the State–Trait Anger Expression Inventory (Spielberger et al., 1999) was used to measure anger. The State Anger scale measures the subjects' mood at the time of the test. These questionnaires can be used to measure the mood of healthy subjects.

#### Other outcome measures

Several questionnaires designed to assess traits or states related to longer time periods were collected but are not described here. For other performance-type psychological cognitive tests used in this experiment, please see our previous study (Takeuchi et al., 2013b).

### Face-matching task (fMRI task)

#### Task procedures

Subjects in the WMT and control groups participated in the fMRI session before and after completing the intervention period. During the fMRI session, subjects performed five blocks of the face-matching task that were alternated with six blocks of a control-matching task involving simple geometric shapes (there was no rest period between blocks). The face-matching task was used to map training-induced changes in negative emotion-related brain activity (Hariri et al., 2002). In each trial, an image of a face portraying anger or fear was presented at the top of a computer screen and was presented at the bottom left and right sides of the screen simultaneously with two additional images of the same face. One of the two bottom images was a face portraying anger, whereas the other face portrayed fear. Subjects were instructed to select one of the faces presented at the bottom of the screen that was identical to the target face (top). Images were presented sequentially per block. Images represented the target affect (angry or fear) and were derived from a standard set of pictures showing facial effects (Kamachi, 2001). In the control-matching task, the faces were replaced with simple geometric shapes, and the subject was instructed to identify the two matching shapes. The subject had to push a button with the first or second finger if he/she selected the bottom left or right shape, respectively.

Four sets of stimuli, each with a duration of 4.0625 s, were presented in a block, with each block lasting 16.25 s Behavioral performance was recorded as the subject's accuracy and reaction time. For a schematic of the task, please refer to the study by Hariri et al. (2002).

#### Development of stimuli in the previous study

The technical details of how the standard set of pictures of facial effects (Kamachi, 2001) were developed are presented in Kamachi (2001) and Tamamiya and Hiraki (2013). As described in a previous study (Tamamiya and Hiraki, 2013), the database contains 4 females and 6 males and 3 pictures of each model displaying each facial expression. The database also contained the results of a preliminary experiment in which 27 young adults evaluated the intensity of facial expressions and classified the expressions into the following seven categories: happiness, sadness, surprise, anger, disgust, fear, and contempt. The results from that study were used to validate each facial expression.

#### Rationale for fMRI task paradigm

The behavioral task used in this study has been widely used to detect brain activity related to negative emotions, and it has become the gold standard for tasks that have this purpose. Increased activation in the relevant brain areas when viewing angry or fearful faces indicates the induction of negative emotions during the task (Hariri et al., 2002; Meyer-Lindenberg et al., 2006). These changes simply occurred when subjects viewed the faces without having to identify the emotions they portrayed; thus, this is an implicit task. The emotion face task differs from the present control task in the complexity of the stimuli; however, there are no reasons to assume that such differences contribute to brain activity. The reason for using geometrical shapes in the control task was not provided in its original description. We speculate that the aim of this task is to detect brain activity in emotion-related areas; however, how the activity was induced and what factors induced the activity may not be relevant. The same principle applies to the present study.

Other types of negative emotions include sadness or disgust, and faces showing these emotions could be used as stimuli. We only focused on anger and fear because of time limitations, and we chose to use the same procedures consistently among studies (Hariri et al., 2002; Meyer-Lindenberg et al., 2006). For further explanations of why we used this task and not a task for assessing emotional regulation, please see Supplemental Discussion.

### Image acquisition and analysis

MRI data acquisition was conducted using a 3-T Philips Achieva scanner. Forty-two transaxial gradient-echo images (*TE* = 30 ms, *FA* = 90°, slice thickness = 3 mm, *FOV* = 192 mm, matrix = 64 × 64) covering the entire brain were acquired at a repetition time of 2.5 s using an echo planar sequence. For the session for negative emotion-related brain activities, 73 functional volumes were obtained. Diffusion-weighted data were acquired using a spin-echo EPI sequence (*TR* = 10293 ms, *TE* = 55 ms, *FOV* = 22.4 cm, 2 × 2 × 2 mm^3^ voxels, 60 slices, SENSE reduction factor = 2, number of acquisitions = 1). The diffusion weighting was isotropically distributed along 32 directions (*b*-value = 1,000 s/mm*^2^*). Using a spin-echo EPI sequence (*TR* = 10293 ms, *TE* = 55 ms, *FOV* = 22.4 cm, 2 × 2 × 2 mm^3^ voxels, 60 slices), three images with no diffusion weighting (*b*-value = 0 s/mm^2^) (*b* = 0 images) were acquired from 52 subjects and one *b* = 0 image was acquired from nine subjects. From the collected images, FA and ADC maps were calculated (Takeuchi et al., 2011c). These calculated FA and ADC images were used for preprocessing. Other imaging data included scans obtained with fMRI during WM, scans for resting state functional connectivity (FC), arterial spin labeling, and T1-weighted structural images, all of which were used in our previous study of WMT (Takeuchi et al., 2013b).

### Preprocessing of functional activation data

Preprocessing and analysis of functional activation data were performed using SPM8 implemented in Matlab. Before their analysis, BOLD images from the preintervention and postintervention scans were realigned and resliced to the mean image of the BOLD images from the preintervention scans. For each subject, the skull and skin appearing in the mean BOLD images and *b* = 0 images were removed through intensity thresholding of the spatially smoothed images, as described previously (Takeuchi et al., 2011b). All BOLD images of each subject were coregistered to a skull/skin-stripped *b* = 0 image using the skull/skin-stripped mean BOLD image. Because the *b* = 0 image was aligned with the FA image and ADC map, the BOLD image, *b* = 0 image, FA image, and ADC map were all aligned.

All images were subsequently normalized using a previously validated two-step new segmentation algorithm of diffusion images and the previously validated diffeomorphic anatomical registration through exponentiated lie algebra (DARTEL)-based registration process (Takeuchi et al., 2013a). The voxel size of normalized BOLD images was 3 × 3 × 3 mm^3^.

For additional details on these normalization procedures and their validity, please refer to our previous study (Takeuchi et al., 2013a). In brief, we used these normalization procedures instead of coregistering BOLD and T1-weighted structural images (followed by normalization of the T1-weighted structural image) to ensure precise normalization. Because of the distortion caused by 3-T MRI, the brain's shape in BOLD and T1-weighted structural images can differ, preventing a precise normalization.

### Individual-level statistical analysis of functional imaging data

A design matrix was fitted to each participant with one regressor for each of the face-matching task conditions (when compared with controlled task conditions) in the preintervention and postintervention scans using the standard hemodynamic response function (HRF). The design matrix weighted each raw image according to its overall variability to reduce the impact of movement artifacts (Diedrichsen and Shadmehr, 2005). Six parameters obtained by rigid body correction of head motion were regressed out using these variances with the regressor. We removed low-frequency fluctuations with a high-pass filter using a cut-off value of 128 s. The individual-level statistical analyses were performed using a general linear model. After estimation, beta images were smoothed (6-mm full-width half-maximum) and taken to the second level or subjected to random-effect analysis.

In individual analyses, we examined changes in activation related to negative emotion (the face-matching task vs. the control-matching task) before and after a 4-week intervention period. The resulting maps for each participant represented changes in brain activity during the face-matching task condition between the preintervention and postintervention periods as well as the preintervention brain activity during the corresponding condition. The resulting data were forwarded to group analysis.

The present fMRI tasks did not include resting states. Thus, we were not able to determine whether neural activity was affected by interactions among training effects (preintervention, postintervention), task (emotional task, control task), or group (WMT, control group). Even if we included the resting state, fMRI paradigms for comparing individual differences, group differences, and training effects have resting-state differences in neural activity and suffer from the same limitations. Therefore, we used the gold standard procedure for this fMRI task and removed the resting states (Hariri et al., 2002). Furthermore, because repeated exposure to the same tasks alone greatly alters fMRI responses (Luauté et al., 2009), changes in the control group should be considered as a baseline in this type of experiment.

### Statistical group-comparison analysis of psychological data

Behavioral data were analyzed using SPSS 16.0 (SPSS Inc., Chicago, IL). Because training-related improvements were our primary interest and the basis of our hypothesis, we compared test–retest changes in the WMT group with those in the control group using one-tailed One-Way analyses of covariance (ANCOVA), which is the analysis method used in previous studies of WMT (Klingberg et al., 2002, 2005; Takeuchi et al., 2011d, 2013b). The difference between preintervention and postintervention measures was used as the dependent variable, preintervention scores were the independent variable, and group (WMT or control) was the fixed factor (*P* < 0.05). We used ANCOVAs instead of repeated-measure ANOVAs to control for the effects of preintervention test scores. Statistical experts strongly recommend using ANCOVA instead of repeated-measure ANOVA in this type of study design (Dimitrov et al., 2003). With randomized designs, ANCOVA can reduce error variance, whereas with nonrandomized designs (or with analyses involving substantial preexisting group differences), ANCOVA can adjust the postintervention test means for preintervention test differences among groups (Dimitrov et al., 2003). One may recommend using postintervention test scores instead of differences between preintervention test and postintervention test measures. However, when the preintervention test scores are included as covariates, the two analyses return the same statistical value. Using two-tailed tests was not appropriate in this context because statistical tests should be performed against the hypotheses tested. In this study, the hypotheses did not involve a training-related reduction in mood states.

The behavioral data (accuracy and reaction time) recorded in the face-matching and control-matching tasks were analyzed with the same design but with two-tailed analyses because we did not expect changes in these measures (see Results for details).

### Statistical group-level analysis of imaging data

In the group-level imaging analysis, we tested for group-wise differences in functional activity changes across the entire brain during the emotional face-matching task (compared with the control-matching task). We performed voxel-wise ANCOVAs with the difference in each measure between preintervention scans and postintervention scans at each voxel as the dependent variable and the preintervention scan value at each voxel as the independent variable. Biological Parametrical Mapping (BPM) (Casanova et al., 2007) implemented in SPM5 made it possible for us to use these voxel-wise ANCOVAs by including images representing regional values as covariates. We used SPM5 because BPM was not designed and it has not been thoroughly tested against SPM8. Analysis was performed using SPM5 and images representing preintervention to postintervention changes in functional activity during the emotional face-matching task and functional activity during the emotional face-matching task in the preintervention scan. One may think that the difference in the number of subjects in each group may lead to significant differences in measures of preintervention to postintevention activation changes. However, because statistical analyses tested for differences in changes between groups and not within groups (followed by assertions that significant differences were observed in only one group but not in the other group), this was not possible.

Regions with significance were inferred using family-wise error-based cluster-level statistics (Friston et al., 1996). Only clusters with *P* < 0.05 after correction for multiple comparisons at cluster size with a voxel-level cluster-determining threshold of *P* < 0.005 (uncorrected) were considered statistically significant. This voxel-level cluster-determining threshold has been used in previous studies (Takeuchi et al., 2010a, 2011b), and the validation study (Hayasaka and Nichols, 2003) showed that this threshold does not cause anticonservativeness. If anything, it appears to lead to more conservative results compared with more stringent voxel-level cluster-determining thresholds.

We also performed ANCOVA (two-tailed) to compare group differences between the mean beta estimates of preintervention to postintervention changes in functional activity with the mean beta estimates of preintervention functional activity in clusters with significant WMT-related changes in whole brain analysis, preintervention difference of behavioral data (reaction time, accuracy) between the face emotion task and the control-matching task, as well as pre to post changes in difference of behavioral data (reaction time, accuracy) between the face emotion task and the control-matching task.

### Correlations between behavioral data and functional activity in significant clusters

Furthermore, to reveal the nature of functional activity in areas with significant WMT-related changes, we investigated the association between functional activity and psychological variables using the preintervention data and multiple regression analyses. The dependent variables were the mean beta estimates of functional activity in the clusters with significant WMT-related changes identified in whole brain analysis described above. Independent variables were age, sex, and each psychological variable (scores of measures of emotional states). Data from 95 subjects were included in this analysis. Among the initial 97 subjects considered for the study (see Subjects subsection), data for one subject were not available because of a metal-related problem (Takeuchi et al., 2014b). The data from another subject were removed because the fMRI data were unsuitable. This reduced the number of subjects to 95. We added the data from the additional 16 subjects who only participated in the cross-sectional experiment because they had completed the same protocols for the preintervention period as the subjects in the present study, which included fMRI scans for negative emotion. Excluding these subjects was statistically harmful because it would have reduced the statistical power. For cross-sectional analysis of the brain images, the number of subjects analyzed to determine the effects of WMT (approximately 60 subjects: 41 subjects in the WMT group and 20 subjects in the control group) was quite small; therefore, additional subjects were included in this analysis.

Finally, we investigated whether training-related variables (changes in the composite score of the four WMT tasks), preintervention to postintervention emotional state changes, and preintervention to postintervention functional activity changes in the clusters with significant WMT-related changes identified above were related.

## Results

### Training data

As described in our previous study, to investigate the effect of WMT on resting-state FC, resting-rCBF, and regional gray matter volume using data from the same subjects (Takeuchi et al., 2013b), subjects in the WMT group completed on average 25.87 sessions (*SD* = 2.18) and at least 17 sessions during the 27-day intervention period. The performance on all four trained WM tasks during the last three training sessions was significantly improved compared with that during the first three training sessions (paired *t*-test, *P* < 0.001). Details of the training data and training-related changes in performance scores on cognitive tests (such as WM tasks) have been described previously (Takeuchi et al., 2013b). WMT task performance significantly improved from the first three training days to the last three training days for all tasks (Takeuchi et al., 2013b). The training-related performance change presented in the previous study is illustrated in Supplemental Table 1. Please note that these behavioral data apply to the training tasks performed during the training period, and no data are available for the control group. Compared with the control group, performance on the untrained verbal and visual WM tasks conducted on the day of the MRI sessions in the WMT significantly improved from preintervention to postintervention, as described previously (Takeuchi et al., 2013b).

### The effect of WMT on emotional states (main psychological analyses of this study)

Compared with the control group, the WMT group showed significantly greater preintervention to postintervention reductions in POMS subscale scores for anger/hostility, depression/dejection, and fatigue/inertia but not for the tension/anxiety, vigor/activity, and confusion/bewilderment subscales. Compared with the control group, the WMT group also showed significantly greater preintervention to postintervention reductions for the STAXI score. The results for the psychological scales are shown in Table 1. In these analyses, the statistical values were not corrected for multiple comparisons, which was the case in some previous studies (Klingberg et al., 2002, 2005). Nevertheless, even when these values were corrected using the false discovery rate (Benjamini and Hochberg, 1995), the effects of WMT on the POMS anger/hostility subscale and the STAXI State Anger scale remained significant (*P* < 0.05, corrected). There were also tendencies toward WMT effects (*P* < 0.1) on the POMS depression/dejection and fatigue/inertia subscales, which is congruent with our hypothesis.

**Table 1:** **Pretest and posttest scores for psychological measures (mean ± s.e.m.)**.

	**WMT**		**Control (non-intervention)**		**Planned contrast**	***P*-value^c^**
	**Pre**	**Post**	**Pre**	**Post**		
POMS^a^ –tension–anxiety	6.15 ± 4.66	6.55 ± 5.47	6.00 ± 4.03	6.95 ± 3.99	WMT < control	0.322
POMS—depression–dejection	4.50 ± 4.77	3.63 ± 4.39	3.47 ± 3.89	4.95 ± 4.03	WMT < control	0.048^*^
POMS—anger–hostility	3.73 ± 4.26	3.35 ± 3.85	2.63 ± 2.67	4.79 ± 4.08	WMT < control	0.009^**^
POMS—vigor–activity	8.28 ± 3.78	7.8 ± 4.54	9.42 ± 3.01	9.11 ± 3.05	WMT > control	0.740
POMS—fatigue–inertia	6.63 ± 4.66	6.18 ± 4.71	7.63 ± 4.34	8.53 ± 4.14	WMT < control	0.050^*^
POMS—confusion–bewilderment	4.85 ± 3.71	4.93 ± 3.66	4.37 ± 3.52	5.42 ± 3.85	WMT < control	0.219
STAXI^b^—state–anger	11.8 ± 3.7	11.1 ± 3.1	11.3 ± 3.3	12.9 ± 3.2	WMT < control	0.007^**^

a *Profile of Mood States*.

b *State–Trait Anger Expression Inventory*.

c *One-Way analyses of covariance with test–retest differences in psychological measures as dependent variables and pretest scores of the psychological measures as covariates*.

* *P < 0.05*.

** *P < 0.01*.

Comparisons with a control group are crucial in this type of intervention study. In the present study, average changes in mood scores were observed in the control group. In particular, the score of the POMS anger/hostility subscale significantly increased in the control group (*P* = 0.044, two-tailed paired-t). However, previous studies have shown that mood worsens in the season in which this experiment was performed. Therefore, the changes observed in the control groups may be expected without any intervention or experimental effects. For more details related to this issue, please see Discussion.

### The effect of WMT on negative emotion-related brain activities (main neuroimaging analyses of this study)

**Figure 2:**
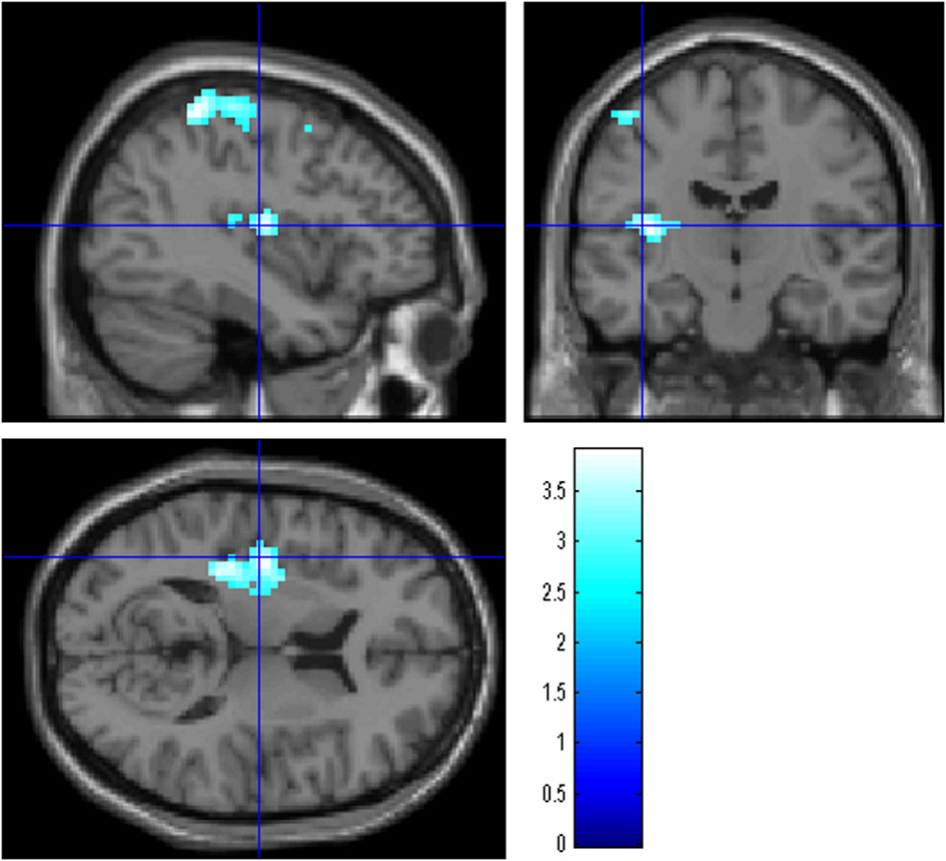
**The effect of WMT on negative emotion-related brain activity in the left posterior insula**. Results are shown with the threshold of *P* < 0.05, corrected for multiple comparisons at the cluster-level with an underlying voxel-level of *P* < 0.005. Findings were overlaid on a “single-subject T1” SPM5 image. Blue represents the T score. Compared with the control intervention (non-intervention), WMT resulted in a significant decrease in functional activity in a cluster spread around the left posterior insula.

**Figure 3:**
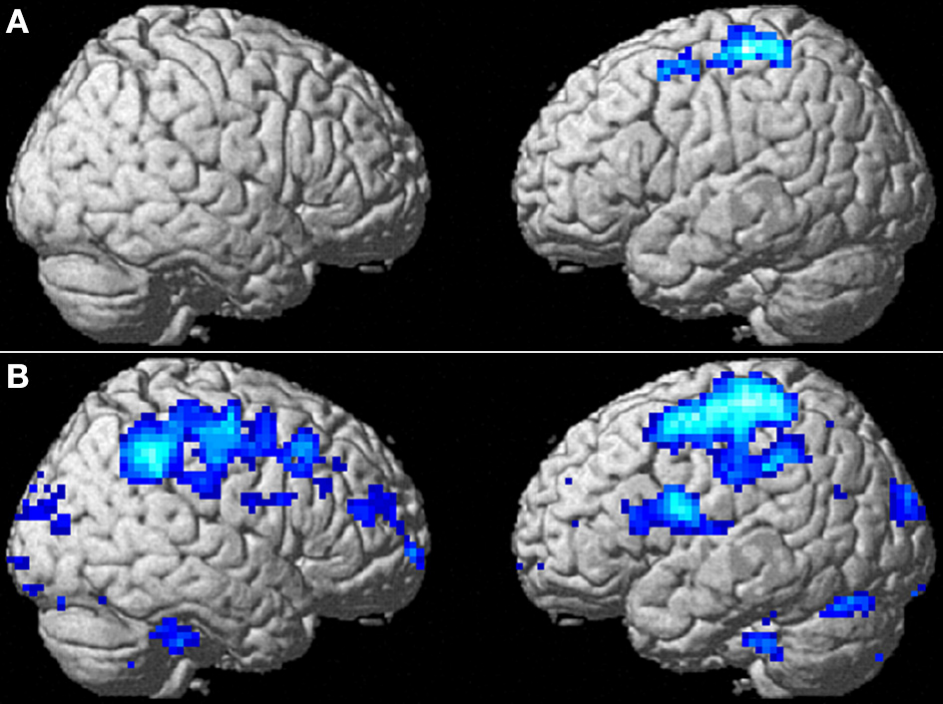
**The effect of WMT on negative emotion-related brain activity in the lateral part of the brain. (A)** Significant results are *P* < 0.05, corrected for multiple comparisons at the cluster level with an underlying voxel level of *P* < 0.005. Blue represents the T score. Compared with the control intervention (non-intervention), WMT resulted in a significant decrease in functional activity in a cluster spread around the left frontoparietal area. **(B)** Tendencies are *P* < 0.05, uncorrected. Compared with the control intervention (non-intervention), WMT resulted in a tendency toward a decrease in functional activity in a cluster spread bilaterally around the extensive frontoparietal areas. Please note that we are not making any conclusions about significant results in the right hemisphere using this second threshold; we are merely showing that laterality of results is unlikely and that no such tendency was observed.

We compared changes in negative emotion-related brain activity in the WMT and control groups. This analysis revealed a statistically significant decrease from preintervention measures to postintervention measures in negative emotion-related activities in an anatomical cluster spread around the left posterior insula (Table 2; Figure 2) and in the anatomical cluster extending from the inferior parietal lobule to the premotor area (Table 2; Figure 3A). The latter cluster slightly extended into Brodmann's area 8, which includes a small portion of DLPFC. The significance of these results was not affected when any preexisting or postintervention group differences in behavioral performance of fMRI tasks were accounted for.

**Table 2:** **Activity related to WMT and negative emotion decreases compared with the control group**.

**Area**		**MNI coordinates of the peak value**			**T score of the peak value**	**Corrected *P*-value (cluster)**
		***x***	***y***	***z***		
Posterior insula	L	−39	−15	12	3.91	0.031
Inferior parietal lobule/premotor area/precentral gyrus/postcentral gyrus	L	−39	−42	60	3.79	<0.001

To determine whether the training-related changes in activity were lateralized to the left hemisphere, we investigated the results using a lenient threshold (uncorrected *P* < 0.05). Please note that we are not making any conclusions about significant results for the right hemisphere using this second threshold; we are merely showing that laterality of the results is unlikely and that no such tendency was observed. This analysis revealed widespread clusters in the frontoparietal area bilaterally and a posterior insula cluster that included the abovementioned significant areas (Figure 3B). These three clusters were significant at *P* < 0.05 after correcting for multiple comparisons at the cluster level with a cluster-determining threshold of *P* < 0.05, uncorrected. Thus, the results may not necessarily be lateralized to the left side.

The *P*-values for comparisons between groups with ANCOVA (two-tailed) for the mean beta estimates for changes in functional activity from preintervention to postintervention with the mean beta estimates of preintervention functional activity in the significant clusters presented in Figures 2, 3A were 0.0001 and 0.00003, respectively. We next performed ANCOVA (two-tailed) that added the following variables (a–d) as covariates using the mean values of each cluster with significant WMT-related changes: (a) accuracy of preintervention face-matching task—accuracy of preintervention control-matching task; (b) [accuracy of postintervention face-matching task—accuracy of postintervention control-matching task]—[accuracy of preintervention face-matching task—accuracy of preintervention control-matching task]; (c) reaction time of preintervention face-matching task—reaction time of preintervention control-matching task; and (d) [reaction time of postintervention face-matching task—reaction time of postintervention control-matching task]—[reaction time of preintervention face-matching task—reaction time of preintervention control-matching task]. *P*-values from ANCOVAs for the clusters presented in Figures 2, 3A were 0.0001 and 0.00002, respectively. These ANCOVA comparisons showed that preexisting group differences in reaction time and accuracy as well as in changes to these behavioral measures did not affect group differences in preintervention to postintervention changes in functional activity for these clusters. We performed these comparisons because covariates (a–d) could not be added to the voxel-by-voxel whole brain analyses because of technical errors in the procedures.

### Behavioral data from the fMRI tasks

The fMRI tasks used in the present study were not cognitively demanding. Therefore, these values were not expected to change, and there is no prior knowledge or theory that suggests individual differences of behavioral performance of this task are even remotely associated with emotion-related cognition. However, preintervetion and postintervention behavioral data for the fMRI tasks are provided in Table 3. ANCOVAs with preintervention to postintervention performance changes in each behavioral data set were used as dependent variables that corresponded to the preintervention performance data as covariates. The group difference as a fixed factor revealed no significant WMT-related effects on either accuracy or reaction time during the tasks.

**Table 3:** **Preintervention and postintervention behavioral data for the fMRI tasks (mean ± s.e.m.)**.

	**WMT**		**Control (non-intervention)**		**Planned contrast**	***P*-value^c^**
	**Pre**	**Post**	**Pre**	**Post**		
Control task RT^a^ (ms)	7767 ± 1294	7872 ± 1337	8299 ± 884	7905 ± 675	Two-tailed^b^	0.087
Emotional face task RT (ms)	12366 ± 2595	12779 ± 2927	12406 ± 1991	12632 ± 1518	Two-tailed^b^	0.754
Control task accuracy (%)	96.5 ± 3.9	96.8 ± 3.0	98.7 ± 2.0	97.6 ± 3.2	Two-tailed^b^	0.640
Emotional face task accuracy (%)	95.1 ± 6.7	94.3 ± 5.7	96.6 ± 3.7	95.8 ± 5.1	Two-tailed^b^	0.471

a *Reaction time*.

b *These tasks are not something one has to perform as much as he or she can; therefore, no training-related improvements in these behavioral data have been assumed*.

c *One-Way analyses of covariance with test–retest differences in psychological measures as dependent variables and preinterveniton scores of the psychological measures as covariates*.

### Correlation between related psychological variables and functional activity in significant clusters before the intervention

To reveal the nature of the functional activity in areas with significant WMT-related changes, we used multiple regression analyses and region of interest (ROI) analysis to investigate the associations between functional activity and psychological variables using data from the preintervention session. In these analyses, the dependent variables were the mean beta estimates of functional activity within the significant clusters presented in Figures 2, 3A, while the independent variables were age, sex, and the individual psychological variables. In particular, we tested whether activity in the posterior insula was associated with the state of anger. We examined data from the 95 subjects (see Subjects subsection and correlations between behavioral data and functional activity in significant clusters subsection of Methods).

**Figure 4:**
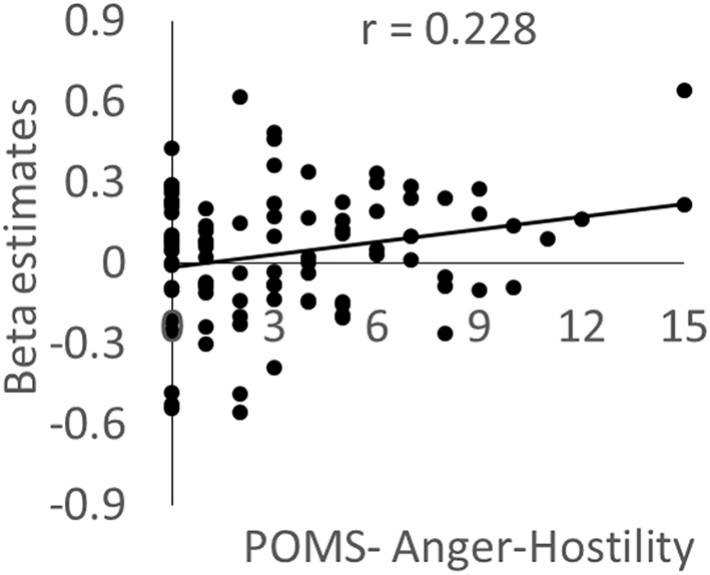
**The association between brain activity related to negative emotions and anger mood state**. Scatterplot showing the relationship between the POMS Anger–Hostility subscale score and the mean value for brain activity during the preintervention period in a cluster in the left posterior insula in which a significant WMT effect was observed.

As expected, activity in the left posterior insula cluster was significantly and positively correlated with the POMS anger/hostility subscale score (*P* = 0.019, *t* = 2.38; effect size *r* = 0.228, Figure 4); however, no significant associations were observed with the STAXI State Anger scale score; there was no evidence of outliers in the scatterplot. This correlation remained significant after excluding the 16 abovementioned subjects who only took part in the cross-sectional experiment (*P* = 0.039). The difference could be due to the higher sensitivity for POMS. No other psychological variables, including each POMS subscale score, showed significant correlations with activity. In addition, no significant correlations were observed when the dependent variable was activity in the left frontoparietal cluster.

The present analyses may appear to be slightly circular in nature because the mean values of clusters with significant WMT effects were used (please note that the same problems occur even when the peak values of clusters with WMT effects are used). However, even when the effects of group assignments were regressed out of our multiple regression analyses by creating and including a covariate of group assignment (WMT group = −1, control group = +1, other subjects = 0), the significance of the correlation between activity in the cluster in the left posterior insula and the POMS anger/hostility subscale score did not change (*P* = 0.030, *t* = 2.20). This shows that the circular nature of the present analysis does not undermine the finding of an association between anger and brain activity in the anterior insula. These results indicate that the increased activity observed during the face-matching task in fMRI was associated with the state of anger.

### Correlations between changes in related psychological variables and changes in functional activity in significant clusters

Following this, we investigated whether training-related variables (changes in the composite score for the four WMT tasks), emotional state changes, and functional activity changes in two of the significant clusters identified above were related. For this analysis, we performed simple correlational analyses between (a) each functional activity change in the two significant clusters and changes in each score of emotional state measures; (b) each functional activity change in the two significant clusters and changes in the composite score for the WMT tasks; and (c) changes in each score of emotional state measures and changes in the composite score for the WMT tasks among subjects in the WMT group. We found no significant correlations; however, the changes in functional activity in the two significant clusters were highly and positively correlated (*P* < 0.001).

There could be a number of reasons for this lack of correlation. One possible reason is insufficient statistical power because we could only analyze data from subjects in the WMT group in this analysis. On the other hand, large individual differences were observed in the control group for emotional state changes, indicating that much of the variance in emotional state changes in WMT group was also unrelated to WMT. In addition, this experiment was not designed for this type of correlation analysis. Although the amount of training is correlated with training-related changes in neural mechanisms (Takeuchi et al., 2010a), we strictly controlled the amount of training performed by subjects in the WMT group (for the actual SD of the number of training sessions, please see Training data subsection of Results). Therefore, little variance was observed among subjects in this respect. To perform these types of correlation analyses successfully, we should not have controlled the amount of training so strictly. Finally, although WMT is effective in decreasing some negative emotional states, WMT is apparently a cognitively demanding and tiring task. Thus, moderate WMT may be effective for improving mood; however, subjects who exert more effort during training may experience an increase in negative feelings during training and consequently have higher scores for negative moods. This may complicate the association between training and mood changes and negate the correlations among WMT-related changes.

## Discussion

In the present study, we investigated whether WMT affected the emotional states and corresponding brain activity in healthy young adults. We observed that individuals who underwent WMT showed decreases in anger, depression, and fatigue as well as decreases in negative emotion-related brain activity in the left posterior insula and in an anatomical cluster near the left frontoparietal area. As discussed below, WMT may reduce anger by increasing the ability to cognitively manage emotional stimuli. WMT may also reduce depressive and fatigue states through previously reported training-induced increases in DLPFC and premotor cortex functioning. These results cannot be entirely explained by preexisting differences between groups because ANCOVA corrected for any preintervention differences. These effects are unlikely to be explained by unknown effects for the non-intervention group, even though the moods in the non-intervention group tended to worsen from the preintervention to postintervention period, as discussed below. The present findings provide new insight into the broader applications of WMT for psychiatric problems, at least among subjects with preclinical-level conditions.

WMT may lead to reduced anger by facilitating cognitive control of situations arousing emotion. Although WMC is not directly related to anger (Hofmann et al., 2008), mental health problems often occur with impaired WMC. For example, substance abuse leads to depression, decreased anger control, suicidal tendencies, and WM deficits (Hoffman et al., 2006). Subjects with a high-functioning WMC are less likely to respond automatically in many situations, including those that provoke emotion (Hofmann et al., 2008). Moreover, they are more likely to correct misapprehensions that lead to anger (Cummins, 2005) and appraise emotional stimuli in an unemotional manner. Thus, they are able to experience and express less emotion in response to such stimuli (Schmeichel et al., 2008). Training-related increases in WMC may lead to decreased anger expression through these mechanisms. Anger and WMC were not correlated despite these previous studies as well as the present study, which may be because anger can lead to arousal and improve performance (Novaco, 1976). Thus, anger and WMC may not reinforce each other through positive feedback.

WMT-related decreases in activity in the frontoparietal region may reflect a decreased cognitive load, which may subsequently allow cognitive management of emotion-inducing stimuli that affect activity in the posterior insula. Numerous studies have shown the posterior insula is associated with pain, anger, and disgust (Calder et al., 2000; Williams et al., 2005; Schultheiss et al., 2008; Paulus et al., 2010). For example, injury to the posterior insula seems to lead to impaired recognition of disgust (Calder et al., 2000). In the present study, subjects performed a face-matching task that involved faces expressing negative emotions, and as they performed the task, activity in the posterior insula was positively correlated with anger/hostility. Thus, the WMT-related decrease in activity in the left posterior insula may reflect a decrease in emotional responses such as anger or other emotions evoked with negative mood-inducing stimuli. On the other hand, the left superior frontal and parietal regions are active during externally directed attention-demanding cognitive tasks (Fox et al., 2005), and this increased activity may generally reflect cognitive load (Takeuchi et al., 2012). Any changes in task-induced activity depends on several factors; and we did not predict the direction of such changes because training-related increases in efficiency (hence an activity decrease) or increase of recruitment of involved areas depend on multiple factors (Erickson et al., 2007). The face-matching task used in the present study appears to have been cognitively demanding because it required additional reaction time. Therefore, the WMT-related decrease in activity in this region may have been related to a decrease in cognitive load during the task. Considering that an increased cognitive capacity allows cognitive management of emotional stimuli, the decreased cognitive load reflected in the frontoparietal area may facilitate cognitive management of emotion-inducing signals that affect posterior insular activity. Alternatively, DLPFC to premotor areas, which are partially included in the frontoparietal area, have been associated with automatic emotion regulation (Mauss et al., 2007). Thus, reduced activity in this area may reflect the load associated with suppression of negative emotion. However, in the present task, the subjects were not required to suppress emotions. Therefore, the nature of task-related activity changes in this area during tasks that induce negative emotion as well as the mechanisms underlying this activity during explicit suppression of emotion remain to be investigated.

It is not clear why after WMT, the frontoparietal area showed activity changes when the extensive DLPFC area that is not overlapped with this area did not. One possibility is a lack of statistical power, considering the right DLPFC showed the same pattern of decreased training-related activity (Figure 3B). Further, training-related structural changes of the bilateral DLPFC (Takeuchi et al., 2013b) have been shown, and DLPFC is involved in the voluntary suppression of emotion (Lévesque et al., 2003).

WMT, like other interventions that stimulate DLPFC, may increase the baseline activity of DLPFC to premotor areas and lead to a concomitant reduction in depressive mood. Patients with depression are characterized by hypometabolism in the cortex, particularly in PFC (Fuster, 2006). Loss of initiative and failure to accurately construct thoughts and conversation are characteristics of depression and are underlain by DLPFC dysfunction (Fuster, 2006). TMS applied to DLPFC to premotor areas can decrease depression and increase resting-state activity in DLPFC to premotor areas (Pascual-Leone et al., 1996; Catafau et al., 2001; Knoch et al., 2006; Johnson et al., 2013). Training on complex divided attention speed tasks, which may well recruit DLPFC to premotor areas because divided attention and speed of complex cognitive tasks are both strongly associated with activity of DLPFC to premotor areas (Thomsen et al., 2004; Takeuchi et al., 2012), also prevents depression (Takeuchi and Kawashima, 2012). These findings suggest that strong cortical stimulation, particularly for DLPFC to premotor areas, leads to increased baseline activity in DLPFC to premotor areas and a concomitant reduction in depressive mood, through TMS or cognitive training. However, the possibility of WMT on clinically depressed subjects remains to be clarified. In the case of abovementioned divided attention speed tasks, the training prevents depression, but the effects of the training on subjects who had already developed depression were less clear (Takeuchi and Kawashima, 2012).

WMT may lead to a reduction in fatigue through training-related activity in DLPFC and its related circuits. It was previously shown that WMT can reduce fatigue in patients with multiple sclerosis (Vogt, 2005). Chronic fatigue syndrome is associated with reduced regional gray matter in the bilateral DLPFC (Okada et al., 2004). The fatigue seems to be related to dysfunction in the frontal–subcortical circuits (Okada et al., 2004). Consistent with this notion, lateral PFC lesions lead to apathy (Fuster, 2006). The present results extend the previous finding of WMT-related reductions in fatigue in patients to healthy young adults, and they show that training-related reductions in fatigue can occur without substantial fatigue and cognitive impairments (Takeuchi et al., 2010b). This change may be caused by the previously identified WMT-induced functional and structural changes in DLPFC (Takeuchi et al., 2010b).

Although the mood states in the control group tended to worsen, it is unlikely that the differences between groups in the present study were caused by effects specific to the control group. The group differences in emotional state changes appeared to be mediated by the average aggregation of emotional states in the control group. However, the present experiments were performed in autumn and winter, and a robust increase in negative mood states, as measured by POMS, has been reported during this period (Harris and Dawson-Hughes, 1993). It is also possible that such changes were strengthened because of distressing events at the university that occurred during winter (such as examinations). Even the patterns of mood changes in this study were similar to those observed in the previous study. Although negative moods increased from autumn to winter in both studies, the increases in depression–dejection and anger–hostility were the most robust, and the reduction in positive mood (vigor) was not as clear. Furthermore, we used the non-intervention group as the control, and because of this simple effect of time-related mood changes, this study design was a strength of our study. If certain meaningful active controls were applied, such as videogame playing, we may not have been able to eliminate the possibility of active control group effects on mood states. Because WMT was limited to less than an hour, it is unlikely that reductions in everyday activities replaced by WMT would have strong effects. Thus, based on the use of a control group in intervention studies and the fact that we used a non-intervention control, it is inappropriate to attribute the group differences in mood change to any specific effect associated with the control group.

There are a few potential pitfalls or limitations to this study. While the state of anger reduction was statistically robust and was confirmed by two different measures, the reduction in fatigue and depression was only marginally significant. These WMT-related reductions in mood are congruent with those observed in previous clinical studies examining WMTs or stimulation applied to DLPFC to premotor areas during cognitive training or TMS; however, these findings warrant further replication.

With regard to the negative findings, no significant WMT-related changes in mood were found for anxiety or other POMS subscales. These findings may be attributed to factors such as a lack of statistical power and the inclusion of outliers. However, anxiety differs from apathy and depression (Fuster, 2006). Psychiatric problems such as anxiety and obsessive–compulsive disorder are associated with hyperactivity in the anterior brain regions (Fuster, 2006; Milad and Rauch, 2007), whereas excessive anxiety may be associated with reduced resting-state brain activity (Gur et al., 1987). In the case of anxiety, these anterior regions include parts of the WM network, such as the dorsal part of the anterior cingulate cortex (Milad et al., 2007). Surgical interventions affecting the dorsal part of the anterior cingulate can attenuate these psychiatric problems, including anxiety (Fuster, 2006; Milad et al., 2007). Functional enhancements in certain parts of the WM network, such as the dorsal part of the anterior cingulate cortex, may not reduce anxiety despite other beneficial effects, e.g., an increased cognitive capacity.

### Conflict of interest statement

The authors declare that the research was conducted in the absence of any commercial or financial relationships that could be construed as a potential conflict of interest.
