# Supplementary material for: Working memory training improves emotional states of healthy individuals
Source: Front Syst Neurosci. 2014 Oct 16;8:200. doi: 10.3389/fnsys.2014.00200 (PMC4199268; doi:10.3389/fnsys.2014.00200)
Supplement: Supplementary file 1 [file Table1.PDF]

Supplemental Table 1 The average of all subjects' highest performances in trained WM tasks among the first and last three training sessions.

	First three sessions (N)	Last three sessions (N)
Visuospatial WM task	8.85 ± 0.78	11.44 ± 1.88
Auditory backward operation span task	8.95 ± 1.44	15.33 ± 3.78
Dual WM task	7.64 ± 0.81	10.10 ± 1.55
Dual N-back task	2.77 ± 0.73	4.92 ± 1.19

Note that the number of training sessions differed for each subject, and therefore, it is difficult to show the average progressive improvement of performance as the number of sessions increased. In all 4 WMT tasks, the highest performance achieved during the last three training sessions by each participant was significantly increased compared with the highest performance achieved during the first three training sessions (paired t-test, $P < .001$).
